# Effects of lentivirus-mediated endostatin on endothelial progenitor cells

**DOI:** 10.18632/oncotarget.21770

**Published:** 2017-10-10

**Authors:** Jing Ai, Jun-Hui Sun, Jian Ma, Ke Yao

**Affiliations:** ^1^ Eye Center, 2nd Affiliated Hospital of School of Medicine, Zhejiang University, Hangzhou 310009, China; ^2^ Key Laboratory of Combined Multi-Organ Transplantation, Ministry of Public Health, The First Affiliated Hospital, School of Medicine, Zhejiang University, Hangzhou 310003, China

**Keywords:** endostatin, endothelial progenitor cells, lentiviral vector, gene transfer, cell viability

## Abstract

Endothelial progenitor cells (EPCs) are candidates for gene therapies against retinal neovascularization (NV). The aim of present study was to investigate the effects of endostatin transfection on EPC function. In the present study, the EPCs were infected with lentivirus overexpressing endostatin. The transfection effects of endostatin overexpression on the proliferation, migratory, differentiation, apoptosis and the cell cycle of this cell line were determined. The real-time quantitative polymerase chain reaction (RT-qPCR) and western blot assays showed high expression levels of endostatin. A cell counting kit-8 assay showed that endostatin overexpression inhibited EPC proliferation. The transwell assay indicated that endostatin overexpression could suppress EPC migration. Furthermore, endostatin overexpression enhanced apoptosis (as showed by AnnexinV-FITC/propidiumiodide staining analysis), induced differentiation and blocked the cell cycle. As compared with negative control group, EPC viability significantly decreased in gene transfection group. In conclusion, present study determined the feasibility of lentivirus-mediated endostatin gene transfer, and indirectly proved the effect of endostatin secretion on EPCs. Also our study provided a new opportunity for the potential application of gene therapy in retinal NV.

## INTRODUCTION

Endostatin is a 20 kDa C-terminal fragment of collagen XVIII with antiangiogenic activity. It specifically inhibits endothelial cell (EC) proliferation and potently inhibits angiogenesis and tumor growth [1-2]. It was reported that endostatin could offer an innovative pharmaceutical strategy for the prevention of retinal neovascularization (NV) [3]. However, a good therapeutic effect requires long-term administration, which is expensive and time-consuming. Endostatin is not stable and its transient effect limits its widespread clinical application. Lentiviral vectors are particularly advantageous and receive much attention due to their stable delivery of the transferred gene into the host cell’s chromosomes [4-6]. Thus, gene transfer strategies [7-12] have the potential to provide sustained, high, local concentrations of antiangiogenic mediators to prevent progression of ocular NV [13-15].

Endothelial progenitor cells (EPCs) have been identified as circulating cells with considerable diagnostic and therapeutic value [16-21]. For example, EPCs can be injected intravitreally into mice with oxygen-induced retinopathy (OIR) to repair the injured retina [16]. Transfected EPCs migrate to the sites of vascular injury to revascularize ischemic tissues [17]. The therapeutic effects of EPCs have been demonstrated in patients with ischemic or NV disease and in autologous EPC populations, representing a novel approach to therapeutic revascularization [18-20]. The genetic engineering of progenitor cells with angiogenic growth factors may be a strategy to enhance the activity of injected progenitors. We hypothesized that EPCs transfected with endostatin could serve as a vehicle for continuous delivery of endostatin to retinal NV tissues.

The present experiment was designed to investigate the potential utility of lentiviral vectors for achieving efficient and stable endostatin expression via gene transfer. The viability of EPCs transfected with the lentivirus-mediated endostatin gene was also investigated.

## RESULTS

### Increased endostatin expression in transfected EPC line

**Figure 1:**
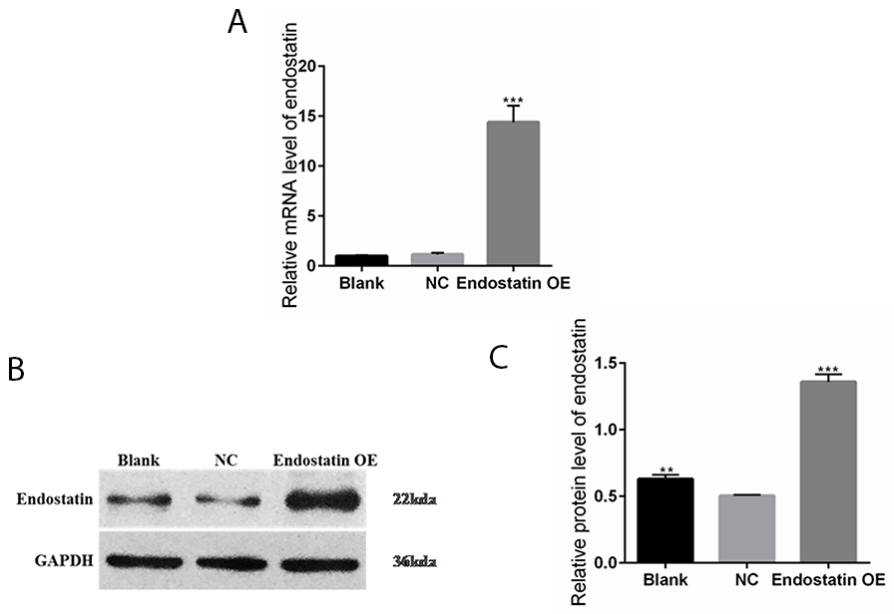
Increased endostatin expression in transfected EPC line Blank: blank control group, NC: negative control group, endostatin OE: endostatin overexpression group. **(A)** Relative mRNA expression levels of endostatin in EPCs detected with qRT-PCR. Compared with NC, ^***^P <0.001. **(B)** Endostatin protein expression levels measured by band intensity analysis. The GAPDH protein served as an internal control. **(C)** The relative protein expression levels of endostatin in EPCs detected by Western blot assays. Compared with NC, ^**^P<0.01, ^***^P<0.001.

Puromycin-resistant individual clones of transgenic endostatin overexpressing EPCs were manually chosen. Total RNA was isolated from the cells in each group. Compared to the NC (negative control EPCs which were transduced with empty vector expressing GFP) group, the endostatin mRNA levels in the endostatin overexpression (OE) group (EPCs which were transduced with recombinant lentiviral vectors expressing both GFP and endostatin) were significantly increased (*P*<0.001); but the endostatin mRNA levels in the blank control (non-transduced EPCs) and NC groups did not increase significantly (Figure 1A).

Western blot analysis (Figure 1B and 1C) demonstrated that endostatin was expressed in the blank control, NC, and endostatin OE groups. Compared to the NC group, the endostatin protein expression levels were significantly increased in the endostatin OE group (*P*<0.001). The endostatin protein expression levels were also significantly increased in the blank control group (*P*<0.01).

### Cell viability

#### Decreased cell proliferation

**Figure 2:**
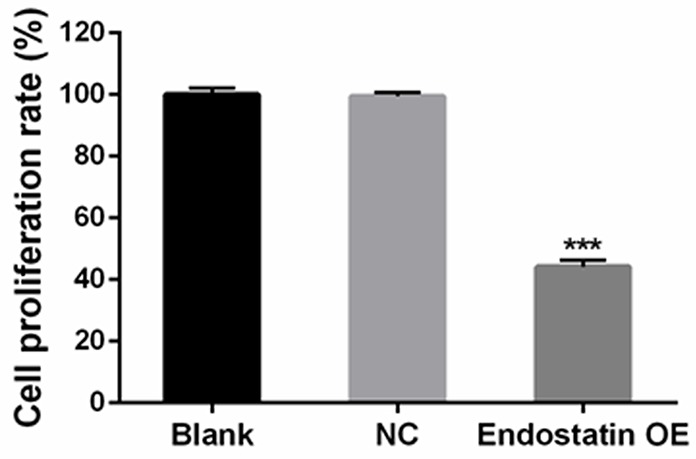
Decreased cell proliferation Cells were seeded in 96-well plates. Effects on cell proliferation ability were analyzed with CCK-8 assays. Blank: blank control group, NC: negative control group, endostatin OE: endostatin overexpression group. Compared with NC, ^***^P<0.001.

Compared to the NC group, the cell proliferation rate was significantly decreased in the endostatin OE group (*P*<0.001). In contrast, there was no difference in the cell proliferation rates between the blank control group and the NC group (Figure 2).

### Decreased cell migration

**Figure 3:**
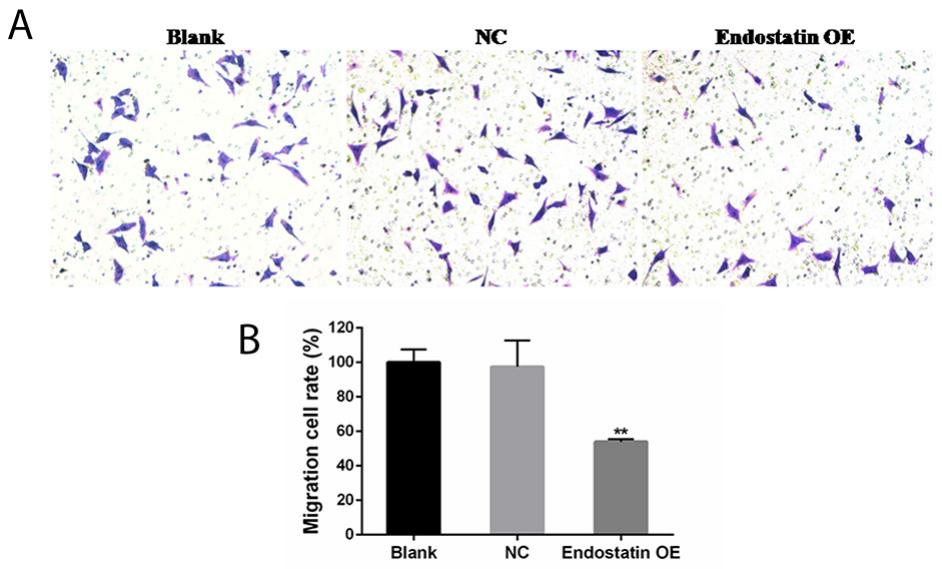
EPC migration inhibited by endostatin overexpression Blank: blank control group, NC: negative control group, endostatin OE: endostatin overexpression group. Compared with NC, ^**^P<0.01. **(A)** EPCs (50,000 per transwell chamber) were seeded in DMEM without FBS in the upper compartment of transwell chambers; lower chambers were filled with DMEM containing 10% FBS. The bottom sides of the filters were stained with crystal violet to count the cells that migrated across the filter. Representative images are shown. Migrating cells were viewed under a microscope (200×). **(B)** Effects of endostatin gene transfer on EPC migration. The graph represents migration cell rate (%) of different groups.

Representative images showed that migrating cells stained with crystal violet in the endostatin OE group were significantly decreased as compared to the blank control and the NC groups (Figure 3A) (*P*<0.01). In contrast, there was no significant difference of EPC migration activity levels between the blank control and NC groups (Figure 3B).

### Restrained cell growth

**Figure 4:**
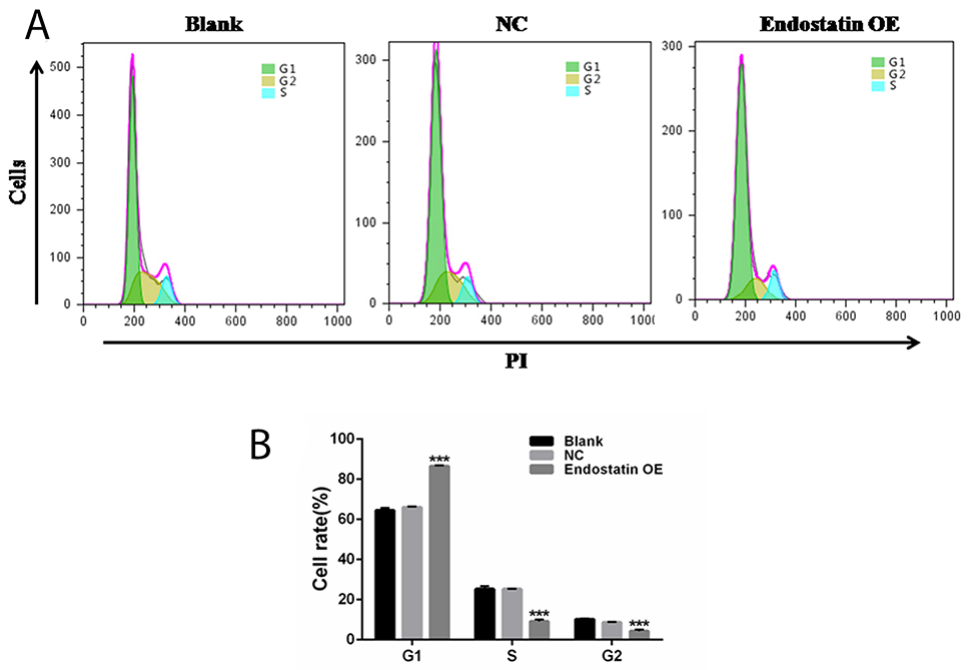
Cell cycle analysis Blank: blank control group, NC: negative control group, endostatin OE: endostatin overexpression group. Compared with NC, ^***^P<0.001. **(A)** Flow cytometry analysis of cell cycle. **(B)** Cell rates of EPCs at different cell cycles. The histogram shows the rates ofthe G_1_, S, and G_2_ phases of the cell cycle for different groups.

Fluorescence-activated cell sorting (FACS) analysis of the cell cycle showed an increased proportion of cells in the G_1_ phase and a decreased proportion of the Sand G_2_ phases in the endostatin OE group compared to the NC group (*P*<0.001) (Figure 4). This indicated that the majority of endostatin-overexpressing EPCs stayed at the G_1_ stage, proving that cell growth was affected and restrained (Table 1, Figure 4). However, there was no difference between the blank control and NC groups (*P*>0.05).

**Table 1:** Percentages of EPCs at different cell cycle phases (%, mean±SD)

Group	G_1_ phase	S phase	G_2_ phase
**Blank**	64.51±1.16	25.25±1.47	10.24±0.31
**NC**	66.01±0.38	25.28±0.15	8.71±0.24
**Endostatin OE**	86.57±0.35^***^	9.14±0.98^***^	4.31±0.79^***^

Note: Blank: blank control group, NC: negative control group, Endostatin OE: endostatin overexpression group. Compared with NC, ^***^P<0.001.

### Increased cell apoptosis

**Figure 5:**
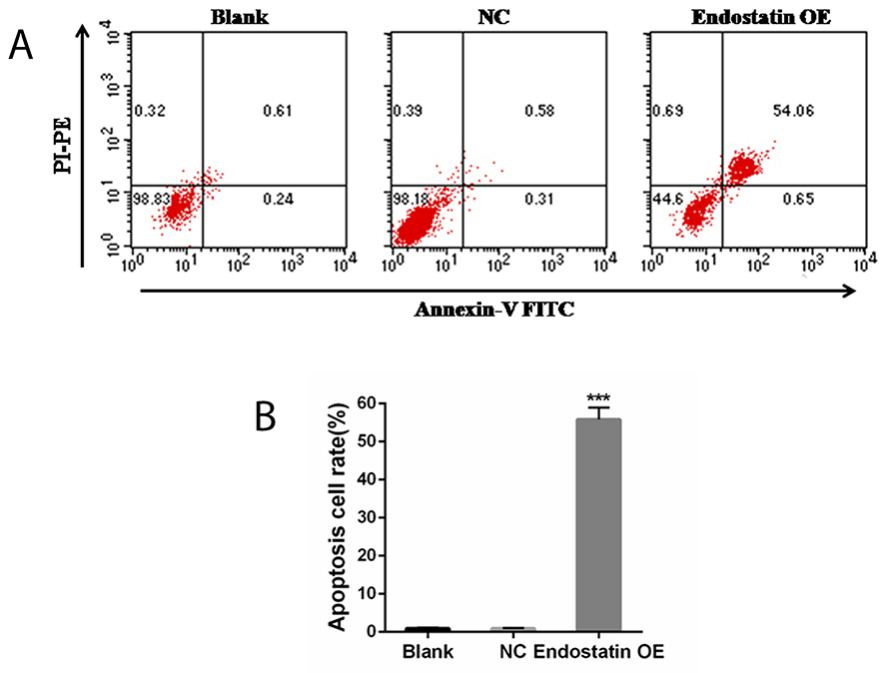
Cell apoptosis analysis Blank: blank control group, NC: negative control group, endostatin OE: endostatin overexpression group. Compared with NC, ^***^P<0.001. **(A)** Flow cytometry analysis of cell apoptosis. PI: propidium iodide, FITC: fluorescein isothiocyanate, PE: phycoerythrin. **(B)** Apoptosis cell rates of different groups (%).

The results of flow cytometry using AnnexinV-FITC/ PI staining indicated that the rate of apoptosis in the endostatin OE group was significantly increased compared to the NC group (*P*<0.001), but there was no difference between the blank control and NC groups (Figure 5).

### Decreased cell differentiation

**Figure 6:**
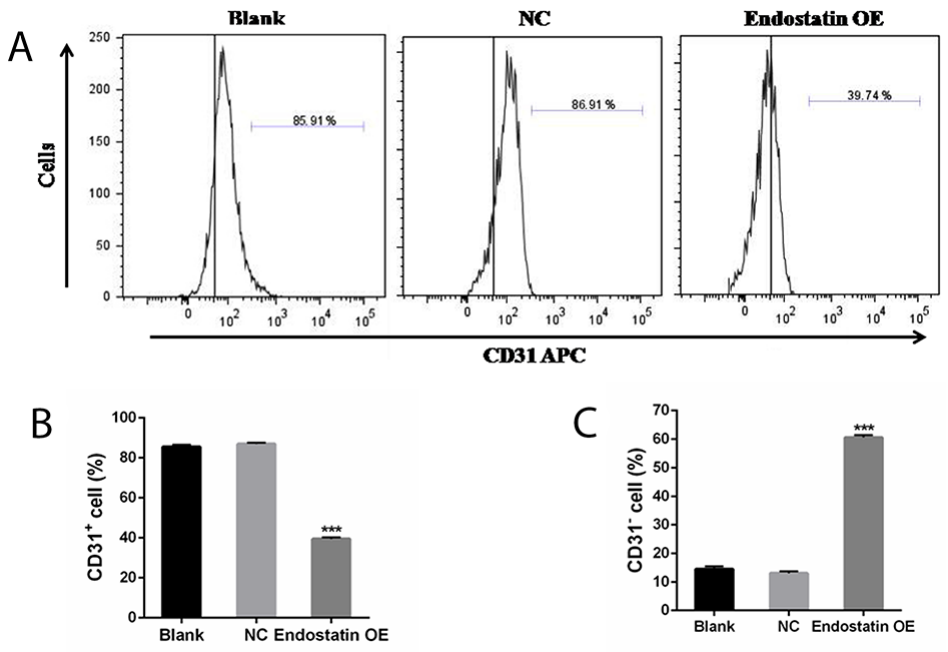
Cell differentiation analysis Percentage of CD31 cells (%). Blank: blank control group, NC: negative control group, endostatin OE: endostatin overexpression group. Compared with NC, ^***^P<0.001. **(A)** Flow cytometry analysis of CD31 APC. **(B)** Percentage of CD31^+^ cells (%). **(C)** Percentage of CD31^−^ cells (%).

The results of flow cytometry, seen in Figure 6, showed that the percentage of CD31^+^ cells was decreased (*P*<0.001), while the percentage of CD31^−^ cells was significantly increased (*P*<0.001) in the endostatin OE group compared to the NC group. As CD31^+^ is a marker of endothelial cell (EC), differentiation in the endostatin OE group was significantly decreased compared to the NC group. There was no difference between the blank control and NC groups (Figure 6).

## DISCUSSION

EPCs are cells of mesodermal origin found in the bone marrow, spleen, umbilical cord blood, and peripheral blood [21]. When NV (angiogenesis or vasculogenesis) occurs, EPCs are mobilized from the bone marrow to the site of NV, and subsequently differentiate into endothelial cells [22-24]. The generation of neovascular-resistant EPCs resulted in the clinical interest, due to the EPC’s therapeutic potential and the EPC’s pathogenic role in NV [21-24]. Endostatin (ananti-angiogenicagent) potently inhibits angiogenesis and has demonstrated anti-tumor effects when delivered continuously [2, 25]. Moreover, lentiviruses have the potential to achieve long-term stable expression and maintain therapeutic levels of secreted peptides [4-6]; therefore, lentivirus-mediated gene delivery of antiangiogenic factors has applications in both functional genomics and clinical studies [26, 27].

In the present study, recombinant lentivirus vectors transfected with endostatin on EPCs were developed to increase long-term endostatin expression. It was observed that EPCs can be genetically modified to overexpress endostatin in a stable fashion. Providing sustained, high, local concentrations of endostatin is possible by increasing endostatin secretion via a gene transfer directly to EPCs.

We investigated the effects of lentiviral-vector-mediated gene transfer of endostatin on the responses of EPCs. It was hoped that endostatin gene transfection of EPCs could enhance EPC’s cell viability, because previous studies [17, 30] reported that gene transfection enhanced EPC’s cell viabilily. For example, transfection of EPCs with the VEGF gene enhances EPC proliferation, adhesion, and incorporation into endothelial cell monolayers [30]. It was suggested that gene delivery combined with EPC transplantation may be a practical and promising therapy for the prevention of neointimal formation after vascular injury. However, the results of the present study contradicted the results of other studies. The results of present study showed that the cellular viability (proliferation, migration, and differentiation abilities) was decreased, the cell cycle was inhibited, and apoptosis was induced in EPCs with endostatin transfection, as compared with control group (EPCs without endostatin transfection). The different results between the present study and other studies may have various causes. First, the functions of transfected genes may be different, and angiogenic factors, such as vascular endothelial growth factor (VEGF), promote proliferation, migration, proteolytic activity, and capillary tube formation in endothelial cells [28, 29]. Hepatocyte growth factor (HGF) can induce endothelial cell proliferation and migration and improve endothelial function [31, 32], while endostatin, as an endogenous inhibitor of angiogenesis, directly affects endothelial cell function by inhibiting proliferation and migration, blocking endothelial cell motility, inducing apoptosis [1, 3, 33], and potently suppressing angiogenesis and tumor growth in animal models [2]. Therefore, endostatin may have some adverse effects on EPCs. Secondly, in the present study, there was no significant difference in cell viability between the NC group (transfected with an empty vector expressing GFP) and the blank control group, suggesting that the gene transfection procedure did not affect EPC viability.

As stated above, cell viability may be affected by endostatin secretion from endostatin-transfected EPCs. However, the results of *in vitro* experiments cannot be applied directly to the human being, therefore, the experimental animal studies are required before the clinical trial. The mechanism of the effects on EPC viability from genes transfected with endostatin should also be investigated.

In conclusion, the present *in vitro* study successfully determined the feasibility of lentivirus-mediated endostatin gene transfer and indirectly proved that endostatin secretion has an effect on EPCs. We expected that the cell viability of endostatin transfected EPCs was increased, because some previous studies reported that EPC viability was enhanced by gene transfection [17, 30]. However, our result showed that the cell viability was decreased. The different results may be due to the different transfected genes [28-29, 31-33]. The transfected genes used in previous studies were VEGF and HGF, which could enhance the cell viability. But the transfected gene used in our study was anti angiogenic factor gene (endostatin), which could inhibit the cell viability. On the other hand, there was a control group (viral transfection of no load gene) in our study, which could determine that the transfection procedure did not influence the cell viability.

## MATERIALS AND METHODS

### Ethics statement

This study received approval from the Institutional Animal Care and Ethics Committee of the 2nd Affiliated Hospital, School of Medicine, Zhejiang University (Permit Number: 2015-012). All methods performed in this study were in accordance with the approved regulations and guidelines.

### EPC isolation and detection

The animals were anesthetized 10 min before each experiment with intraperitoneal injections of sodium pentobarbital (Sigma Aldrich Corp.; St. Louis, MO, USA) at 30 mg/kg. EPCs were isolated and detected according to our previous report [34]. Briefly, to obtain peripheral blood mononuclear cells (PBMCs), blood samples from 250 g Sprague-Dawley rats (Laboratory Animal Center of Zhejiang University) were isolated with Ficoll-PaquePlus (GE Healthcare, USA) and centrifuged by density gradient centrifugation. The PBMCs were then plated in endothelial growth medium (EGM-2-MV; Lonza, Basel, Switzerland) on fibronectin-coated culture dishes. After 96 h, the unattached cells were removed by washing with phosphate-buffered saline (PBS). The cultured medium was replaced every two days thereafter, and each colony was observed. After seven days in culture, the early EPCs were recognized as attached cells with a spindle-shaped morphology. The adherent cells were incubated with 1,1-dioctadecyl-3,3,3’,3’-tetramethylindocarbocyanine-labeled acetylated low-density lipoprotein (Dil-Ac-LDL; #L3484; Thermo Fisher; Waltham, MA, USA) and then fixed in 2% paraformaldehyde for 20 min and counterstained with fluorescein isothiocyanate (FITC)-labeled lectin from ulexeuropaeus agglutinin (UEA-1) (Abcam, Cambridge, UK). Images were acquired under fluorescent microscopy (Motic, China). Cells were also characterized by expression of CD34, CD133, CD31, and Flk-1/VEGFR2. The primary antibodies, anti-CD31 (Abcam, Cambridge, UK), anti-CD34 (Santa Cruz, Dallas, TX, USA), anti-CD133 (Proteintech, Chicago, IL, USA), and anti-CD31 (Abcam, Cambridge, UK) were utilized.

### Recombinant lentivirus construction and transduction of EPC line

The endostatin fragment was amplified with polymerase chain reaction (PCR). The upstream and downstream primers, respectively, were as follows: B2085CEF 5′-AGGGTTCCAAGCTTAAGCGGCCGCGCCACCATGCATACTCATCAGGACT-3′ and B2085CER 5′-ATCAGTAGAGAGTGTCGGATCCTTATTTGGAGAAAGAGGTCATGAAG-3′. The clone was between the Not I and BamH I restriction sites. The transfections with the lentiviral (LV) transfer construct (LV5-EF1a-GFP+PURO) encoding green fluorescent protein (GFP) (Shanghai Gene Pharma Co., Ltd., China) were performed by lipofection for stable expression. Briefly, 293T cells were transfected with plasmid pLV/helper-SL3, pLV/helper-SL4, pLV/helper-SL5, and endostatin DNA, using Lipofectamine® 2000 reagent (Invitrogen, Grand Island, NY, USA) according to the manufacturer’s instructions. Recombinant lentivirus (LV5-EF1a-GFP+PURO-endostatin) was collected from 48 to 72h after transfection, concentrated by low-speed centrifugation at 3,000 *g* for 15 min, and filtered through a 0.45 μm filter. The viral supernatant was concentrated by ultracentrifugation at 50,000 *g* for 90min and stored at -80°C.

For EPC transduction, the cells were re-suspended in Dulbecco’s minimum essential medium (DMEM) (HyClone, USA) at a density of 1×10^6^cells per 6-well plate, then cultured for 24h. Recombinant lentiviral vectors (LV5-EF1a-GFP+PURO-endostatin) were diluted at a multiplicity of infection (MOI) of 100 in DMEM supplemented with 10% fetal bovine serum (FBS) and in the presence of 5μg/ml polybrene (5μg/ml; Sigma, USA). Transduction was performed in round-bottomed 6-well plates for 24 h. After puromycin (1μg/ml) selection and four consecutive days of culturing, stable transduction of the EPC line was obtained. Cells that were non-transduced and those that were transduced with an empty vector expressing GFP were used as the blank control and negative control (NC) groups.

### RNA extraction and reverse transcription-quantitative PCR (RT-qPCR) assays

Total RNA was extracted from the transfected EPCs using TRIzol reagent (Invitrogen, USA) following the manufacturer’s protocol. The purity of the RNA preparation was verified by spectrophotometry readings with a Nano Drop instrument (Thermo Scientific, USA) and the integrity of the extracted RNA was evaluated by separation on agarose gel. Reverse transcription was carried out with a PrimeScript®RT reagent Kit (Perfect Real Time; Takara, Dalian, China). The primers for each gene were as follows: endostatin Fwd (5-TCTCCCAAGTCGAAGACCCT-3) and endostatin Rev (5-GAACAGCAGCGAAAAGTCCC-3); VEGF Fwd (5-GTGAGCCTTGTTCAGAGCG-3) and VEGF Rev (5-GACGGTGACGATGGTGG-3); GAPDH Fwd (5-TCTCTGCTCCTCCCTGTTCT-3) and GAPDH Rev (5-ATCCGTTCACACCGACCTTC-3). Amplification was carried out as follows: 95°C for 3min; 40 cycles at 95°C for 12 sec, and 62°C for 40 sec. All reactions were run in triplicate and the 2^-ΔΔCt^ equation was used to analyze the relative gene expression of endostatin and VEGF, with GAPDH as the endogenous reference.

### Western blot

Whole cells were lysed and protein samples containing 40 μg of protein were separated on 10% sodium dodecyl sulfate-polyacrylamide gels (SDS-PAGE), then electro-transferred onto polyvinylidene fluoride (PVDF) membranes (MilliporeCorp., Bedford, MA, USA). They were then blocked in TRIS-buffered saline containing Tween 20 (TBST; China National Pharmaceutical Group Corporation; Beijing, China) and 5% nonfat milk for 2 h to block nonspecific binding, then rinsed with TBST. The blots were incubated with primary first antibodies to endostatin (1:1000; Abcam, UK), VEGF (1:1,000; Proteintech, USA), and GAPDH (1:2000; MultiSciences, Hangzhou, China) at 4°C overnight. After the membranes were washed several times with TBST, appropriate horseradish peroxidase (HRP)-conjugated secondary antibodies (1:20,000; MultiSciences, Hangzhou, China) were added for 2 h at room temperature. Finally, the membranes were washed, and the enhanced chemiluminescence (ECL) substrates were detected (GE Healthcare, USA). GAPDH was used as a loading control. Data were analyzed with Gel-Pro Analyzer software.

### Cell-counting kit-8 (CCK-8) cell proliferation assay

Cell proliferation was detected with CCK-8 (SAB; College Park, MD, USA) according to the manufacturer’s instructions. In brief, cells were seeded in 96-well plates at a density of 3×10^4^ cells/mL, and cultured at 37°C and 5% CO_2_ overnight. Next, 100μl of CCK-8 solution reagent was added to each well (10 μl CCK-8 and 90 μl DMEM). The plates were incubated for an additional 1h at 37°C and 5% CO_2_. The absorbance at 450nm was measured with a microplate reader (DNM-9606; Beijing Prolong Co., Ltd., Beijing, China).

### Transwell assay

A transwell chamber (Corning Life Sciences, Tewksbury, MA, USA) was placed into a 24-well plate. Cells in each group were digested with trypsin and re-suspended in serum-free DMEM, then 0.5 ml of cells were added to the upper chamber (1×10^5^ cells/mL) while 0.75 ml DMEM containing 10% FBS was added to the lower chamber. After incubation at 37°C for 48 h, the cells that had not migrated were removed with a cotton swab. The migrating cells in the lower chamber were fixed with 1ml of 4% paraformaldehyde (Solarbio, Solarbio® Life Sciences, Beijing, China) at room temperature for 30 min and stained with crystal violet (Solarbio, Solarbio® Life Sciences, Beijing, China) staining solution for 1 h. Five fields were randomly selected and the number of migrating cells was counted under an Olympus optical microscope (Japan).

### Cell cycle analysis

The cell cycles were analyzed with flow cytometry (BD Biosciences, Franklin Lakes, NJ, USA) using the data acquisition Modfit software. Briefly, after transfection, cells were trypsinized, collected into Eppendorf tubes, washed with PBS by centrifugation at 1,000 *g* for 5 min, and fixed in 70% ethanol at 4°C overnight. Cells were then centrifugated, washed with PBS, and incubated with 10 uL RNase A (20 ug/mL; Sigma, Aldrich Corp., St. Louis, MO, USA) for 30 min at 37°C. They were washed again with PBS and stained with propidium iodide (PI; BD Biosciences, Franklin Lakes, NJ, USA) and TritonX-100 (Solarbio, Solarbio® Life Sciences, Beijing, China) at room temperaturein the dark. PI fluorescence was then analyzed with flowcytometry.

### Cell apoptosis analysis

The cells were trypsinized, counted, and seeded. Later, the cells were harvested and stained with AnnexinV-fluorescein isothiocyanate (FITC)/PI according to the manufacturer’s instructions. Briefly, the cells were washed twice with PBS and re-suspended in binding buffer. They were subsequently incubated with 5 μl of AnnexinV-FITC (BD Biosciences, Franklin Lakes, NJ, USA) and 5 μl of PI at room temperature in the dark for 15 min, then analyzed with flow cytometry.

### Cell differentiation analysis

For differentiation, the cells were trypsinized, collected, washed with 0.01M PBS, and fixed with 4% paraformaldehyde for 20 min, then centrifugated at 1,000 *g* for 5 min. The cells were then washed with 0.01M PBS and incubated with APC-CD31 antibody (Abcam, Cambridge, UK) at 4°C for 30 min. They were then analyzed with flow cytometry.

### Statistical analyses

SPSS19.0 (SPSS, USA) software was used for statistical analyses. Standard deviation (SD) and average values were calculated for each variable (data are mean±SD). Groups were compared usingone-way analysis of variance (ANOVA). A P-value of <0.05 indicated a statistically significant difference. Each *in vitro* experiment was repeated independently at least three times.

## Abbreviations

EPCs: Endothelial progenitor cells
NV: neovascularization
OIR: oxygen-induced retinopathy
VEGF: vascular endothelial growth factor
HGF: Hematopoietic growth factor
GFP: green fluorescent protein
PBMCs: peripheral blood mononuclear cells
FITC: fluorescein isothiocyanate
PI: propidiumiodide
UEA: ulexeuropaeus agglutinin
